# Current status and perspective of tumor immunotherapy for head and neck squamous cell carcinoma

**DOI:** 10.3389/fcell.2022.941750

**Published:** 2022-08-26

**Authors:** Chenhang Yu, Qiang Li, Yu Zhang, Zhi-Fa Wen, Heng Dong, Yongbin Mou

**Affiliations:** ^1^ Nanjing Stomatological Hospital, Medical School of Nanjing University, Nanjing, China; ^2^ Department of Clinical Laboratory, Nanjing Maternity and Child Health Care Hospital, Women’s Hospital of Nanjing Medical University, Nanjing, China

**Keywords:** immunotherapy, head and neck cancer, squamous cell carcinoma, immune escape, oral squamous cell carcinoma

## Abstract

Head and neck squamous cell carcinoma (HNSCC) have a high incidence and mortality rate, and investigating the pathogenesis and potential therapeutic strategies of HNSCC is required for further progress. Immunotherapy is a considerable therapeutic strategy for HNSCC due to its potential to produce a broad and long-lasting antitumor response. However, immune escape, which involves mechanisms including dyregulation of cytokines, perturbation of immune checkpoints, and recruitment of inhibitory cell populations, limit the efficacy of immunotherapy. Currently, multiple immunotherapy strategies for HNSCC have been exploited, including immune checkpoint inhibitors, costimulatory agonists, antigenic vaccines, oncolytic virus therapy, adoptive T cell transfer (ACT), and epidermal growth factor receptor (EGFR)-targeted therapy. Each of these strategies has unique advantages, and the appropriate application of these immunotherapies in HNSCC treatment has significant value for patients. Therefore, this review comprehensively summarizes the mechanisms of immune escape and the characteristics of different immunotherapy strategies in HNSCC to provide a foundation and consideration for the clinical treatment of HNSCC.

## 1 Introduction

Head and neck squamous cell carcinoma (HNSCC) is a type of solid tumor that develops from the mucosal epithelium of the mouth, pharynx, and larynx (Solomon et al., 2018; Kitamura et al., 2020; Ding et al., 2021). HNSCC, as the sixth most common cancer in the world, results in an annual death toll of approximately 350,000 and a 5-years morbidity and mortality rate of approximately 40%–50%. HNSCC of the oral cavity (oral squamous cell carcinoma, OSCC) and larynx is frequently linked to tobacco, alcohol, and betel nut use, whereas HNSCC of the oropharynx is mostly linked to human papillomavirus (HPV) infection (Kawakita and Matsuo, 2017; McDermott and Bowles, 2019a; McDermott and Bowles, 2019b; Li et al., 2020; Zhou and Parsons, 2020). Currently, the main treatment option for HNSCC is a combination of surgery, chemotherapy, and radiotherapy. However, the prognosis of HNSCC patients remains poor due to late diagnosis, high rates of primary-site recurrence, and lymphatic metastasis. Thus, there has been no significant improvement in long-term patient survival. The treatment of recurrent/metastatic HNSCC is one of the most difficult clinical challenges.

**FIGURE 1:**
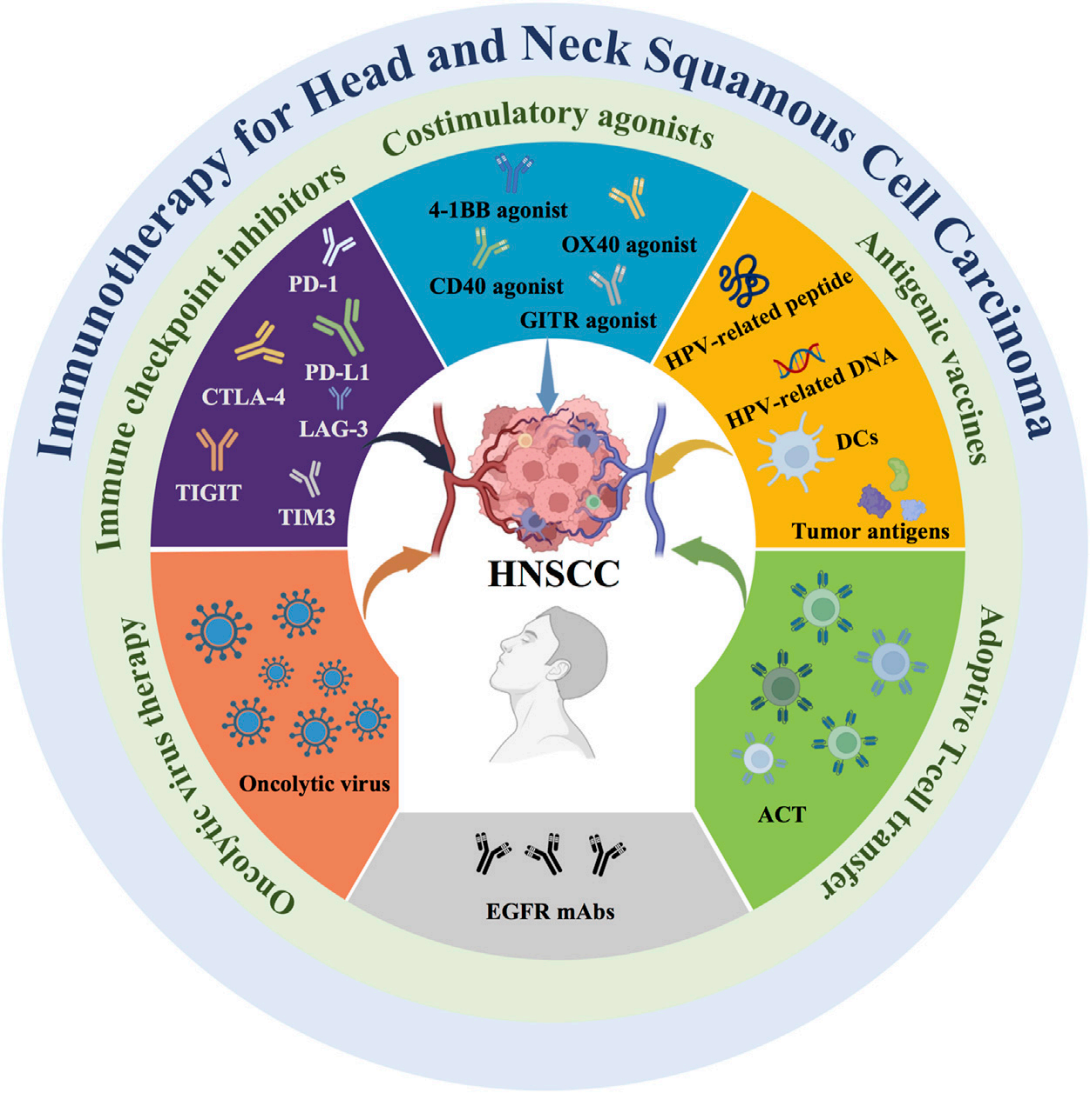
Current immunotherapy strategies for HNSCC. Current immunotherapies for HNSCC include immune checkpoint inhibitors, costimulatory agonists, antigenic vaccines, oncolytic virus therapy, adoptive T cell transfer (ACT), and epidermal growth factor receptor (EGFR)-targeted monoclonal antibodies (mAbs).

Emerging tumor immunotherapy is an important method for the treatment of HNSCC (Huang et al., 2022a). Activating an effective immune response can impair the phenotype and function of tumor cells, killing malignant cells, and trigger an adaptive immune response (Xia et al., 2021). However, immune escape caused by multiple mechanisms in HNSCC limits the immune system from recognizing and attacking tumor cells (Wang et al., 2021a). Therefore, immunotherapies overcoming the immune escape and enhancing immune killing has become an important goal (Zhang and Zhang, 2020). Current immunotherapy takes many forms (Figure 1), including immune checkpoint inhibitors, costimulatory agonists, antigenic vaccines, oncolytic virus therapy, adoptive T cell transfer (ACT), and epidermal growth factor receptor (EGFR)-targeted therapy. On the one hand, these immunotherapies target a variety of coinhibitory and costimulatory signaling molecules present on the surface of immune cells. The application of immune checkpoint inhibitors and costimulatory agonists can achieve effective antitumor immune effects. On the other hand, antigenic vaccines are a type of active immunotherapy in which antigens are derived from tumors. Antigenic vaccines delivered to the immune system in a sufficiently immunogenic context can elicit an antitumor immune response against tumor-associated antigens (TAAs) and tumor-specific antigens (TSAs) (Melief et al., 2015). In addition, oncolytic virus therapy is based on the direct killing effect of viruses on tumor cells to achieve antitumor effects. ACT requires taking tumor-specific T cells from patients and expanding them *in vitro* and then infusing them back into the patient to kill the tumor cells. Therefore, oncolytic virus therapy and ACT are two promising immunotherapy approaches due to their specific therapeutic effects.

Herein, we summarize the immune escape mechanisms and review existing immunotherapeutic strategies for HNSCC. In addition, the advantages and limitations of different immunotherapy strategies are further discussed to provide theoretical support for clinicians.

## 2 Immune escape mechanisms of HNSCC

**FIGURE 2:**
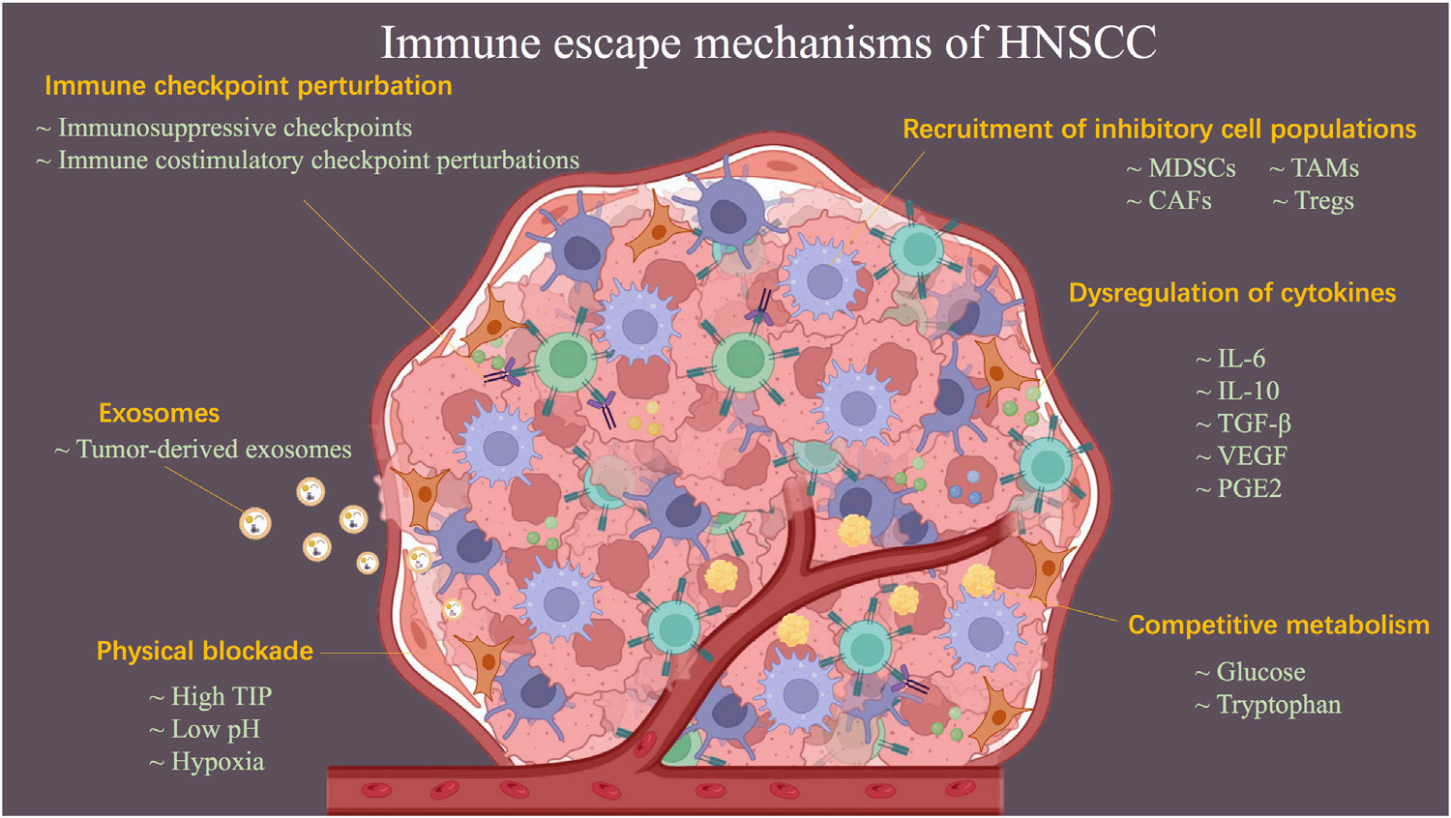
Schematic diagram describing the mechanisms of HNSCC-evaded immunity. Immune escape mechanisms, including physical blockade, dysregulation of cytokines, perturbation of immune checkpoint, recruitment of inhibitory cell populations, negative influences of exosomes, and competitive metabolism of tumor cells, result in HNSCC becoming a refractory tumor.

Ideally, the immune system would be able to exert an active immune killing effect by recognizing TAAs or TSAs. However, HNSCC is characterized by a combination of immune escape mechanisms to suppress immune attacks, which results in low response rates to immunotherapy in the clinical management of HNSCC-related conditions. In the following, the mechanisms of HNSCC-evaded immunity are discussed, including physical blockade, dysregulation of cytokines, perturbation of immune checkpoints, recruitment of inhibitory cell populations, negative influences of exosomes, and competitive metabolism of tumor cells (Figure 2).

### 2.1 Physical blockade of immune cell infiltration

The elevated tumor interstitial pressure (TIP) physically blocks the infiltration of immune cells. On the one hand, the high rate of proliferation of HNSCC cells leads to the formation of abnormal blood vessels and lymphatic vessels, allowing fluid to leak and accumulate further in the interstitium. On the other hand, in the HNSCC tumor microenvironment (TME), the stiff and swollen extracellular matrix (ECM) combines with rapidly proliferating cancer cells, further leading to an elevated TIP (Ariffin et al., 2014). The unstable vascular system of tumors helps maintain a TME characterized by a low pH, hypoxic environment in which compounds such as galactose lectin-1 and adenosine, which inhibit effector T cells (Teff), are induced to be secreted, ultimately creating a permissive environment for tumor growth (Schaaf et al., 2018). In addition, tumor extracellular matrix induced drug resistance and immune suppression *via* high expression of collagen (Peng et al., 2020).

### 2.2 Dysregulation of cytokines

When a proportion of immune cells overcome the barrier of high TIP and infiltrate into the nest of HNSCC tumor cells, dysregulation of many cytokines in HNSCC can mediate immune escape. Transforming growth factor (TGF)-β is a regulatory cytokine, and overexpression of TGF-β1 in HNSCCs has been reported to lead to over proliferation of head and neck epithelial cells and then enhance inflammation and angiogenesis (Lu et al., 2004). Moreover, TGF-β has been shown to promote tumor progression in HNSCC through mechanisms, such as impeding dendritic cell (DC) maturation (Wrzesinski et al., 2007), inhibiting natural killer (NK) cytotoxic effects (Klöss et al., 2015), and affecting TME-associated immunosuppressive cells (Pang et al., 2018).

Furthermore, interleukin (IL)-6 and IL-10 signaling have tumor-promoting functions. IL-6 induces tumor pro-survival and pro-proliferation signaling by activating signal transducers and activators of transcription (STAT) 3 (Shinagawa et al., 2017), and promotes tumor immune escape by further inhibiting the activation of DCs, NKs, and T cells (Cheng et al., 2003). IL-10 can promote immune suppression by inhibiting interferon (IFN)-α secretion by plasmacytoid dendritic cells (pDCs) (Bruchhage et al., 2018). In addition, vascular endothelial growth factor (VGEF) and prostaglandin (PG) E2 are upregulated in HNSCC tumor cells. The former inhibits the maturation of DCs and leads to inactivation of T cells (Siemert et al., 2021). The latter can suppress immune responses mediated by adaptive regulatory T cells (Balch et al., 1982).

### 2.3 Perturbation of immune checkpoints

There are two types of immune checkpoints: immunosuppressive checkpoints and immune costimulatory checkpoints. In healthy humans, immunosuppressive checkpoints control the immune response. They are often expressed on immune cells (e.g., T cells), and when bound to the corresponding ligand, they effectively suppress the immune response, thus protecting the body from autoimmune diseases (Sharma and Allison, 2015). In contrast, in HNSCC, tumor cells intelligently exploit the specific physicochemical properties of the TME to mediate the upregulation of immunosuppressive receptors or ligands, ultimately inhibiting the antitumor effects of the immune system (Deng et al., 2018). Several immunosuppressive checkpoints have been reported to be associated with HNSCC, including programmed death-1 (PD-1), programmed death ligand-1 (PD-L1), cytotoxic T-lymphocyte-associated protein-4 (CTLA-4), lymphocyte activation gene-3 (LAG-3), T cell immunoglobulin mucin-3 (TIM-3), and T cell immunoglobulin ITIM domain (TIGIT) (Moy et al., 2017; Deng et al., 2018). Perturbations of the immune costimulatory checkpoints have also been reported in HNSCC. The costimulatory signal is a secondary signal that mediates the activation and proliferation of immune cells (Sanchez-Paulete et al., 2016; Liao et al., 2019). Important costimulatory signaling molecules that have been identified in HNSCC include CD137, OX40 (CD134), CD40, and glucocorticoid-induced tumor necrosis factor receptor (GITR) (Liao et al., 2019). However, the specific TME of HNSCC accomplishes immune escape by blocking the enrichment and activation of costimulatory signals.

### 2.4 Recruitment of inhibitory cell populations

Relevant immunosuppressive cells in the TME of HNSCC mainly include T regulatory cells (Tregs), myeloid-derived suppressor cells (MDSCs), tumor-associated macrophages (TAMs), and cancer-associated fibroblasts (CAFs) (Davis et al., 2016). Tregs are a heterogeneous population of T cells with immunosuppressive functions. Recruitment of Tregs to the TME occurs by binding of C-C chemokine receptor type 4 (CCR4) on the T cell surface by macrophage-derived chemokine (MDC/CCL22) produced by the tumor (Nishikawa and Sakaguchi, 2014; Propper and Balkwill, 2022). In addition, Tregs activate angiogenic markers such as vascular endothelial growth factor (VEGF) in the TME, which promotes tumor angiogenesis (Lugano et al., 2020). Moreover, Tregs mediate tumor immune escape through the production of TGF-β and IL-10 (Dennis et al., 2013; Batlle and Massagué, 2019).

MDSCs are a heterogeneous group of immature myeloid cells that can significantly suppress the immune cell response. In the TME of HNSCC, MDSCs highly express arginase 1 (Arg-1), inducible nitric oxide synthase (iNOS). L-arginine is a common substrate for Arg-1 and iNOS, and thus L-arginine in the TME is continuously depleted. This ultimately affects the maturation of T cell receptors (Szefel et al., 2019). High concentrations of ROS also inhibit T cell responses (Ohl and Tenbrock, 2018); in parallel, MDSCs promote the production of Tregs (Serafini et al., 2008).

Mature macrophages become TAMs upon recruitment to the TME of HNSCC, and TAMs use receptors (e.g., Gas6 and Protein S) as bridging ligands to bind to the “eat-me” signal phosphatidylserine on the apoptotic cell membrane, further polarizing TAMs to the protumor M2 phenotype (Myers et al., 2019). M2 TAMs mediate tumor immunosuppression through the secretion of immunosuppressive cytokines such as IL-1β, IL-6, IL-10, and TGF-β and suppress T cell immune responses through the expression of the immunosuppressive ligand PD-L1 (De Palma and Lewis, 2013).

CAFs are supportive stromal cells in the TME and are involved in the remodeling of the extracellular matrix, and the cancer nest is thus protected by the stroma. In HNSCC, CAFs have been shown to inhibit T cell proliferation *via* the PD-1/PD-L1 axis (Takahashi et al., 2015). Furthermore, it was reported that CAF-derived lactate enhances HNSCC tumor development by increasing oxidative phosphorylation (OXPHOS) activity (Jiang et al., 2019).

### 2.5 Negative influences of exosomes

Exosomes are biologically active and informative bilayer nanovesicles (30–150 nm) derived from the endosomal pathway. Tumor-derived exosomes (TDEs) consist mainly of abundant bioactive proteins (oncoproteins, immunomodulatory molecules, and growth factors, etc*.*) and nucleic acids (microRNA, mRNA, etc*.*) that convey information (Huang et al., 2022b). There is growing evidence that exosomes are directly related to HNSCC-mediated immune escape (Wang et al., 2021b).

Exosomes influence the growth and progression of HNSCC. Angiogenesis is an important factor in promoting tumor proliferation. A mouse model study confirmed that TDE promotes tumor angiogenesis through phenotypic regulation of endothelial cells, which may contribute to the growth and metastasis of HNSCC (Ludwig et al., 2018). Another study found that TDEs containing TGF-β promoted HNSCC angiogenesis and drove tumor progression (Ludwig et al., 2020). In addition, some studies have shown that RNA from TDEs, plays an important role in promoting HNSCC progression and metastasis. CAFs were reported to promote the proliferation and metastasis of tumor cells by transferring exosomal miR-34a-5p (Li et al., 2018) and miR-382-5p (Sun et al., 2019) into them. Exosomes derived from hypoxic tumor cells can promote cell migration and invasion by delivering miR-21 to normoxic HNSCC cells (Wang et al., 2016).

Exosomes can also contribute to an immunosuppressive microenvironment. First, exosomes can mediate immunosuppression by altering the number and activity of immunosuppressive cells, such as Tregs and MDSCs (Xiao et al., 2019). A recent animal study demonstrated that exosomal CMTM6 from tumor cells induced polarization of M2 TAMs (Pang et al., 2021). Furthermore, exosomes secreted from HNSCC are enriched with suppressive molecules such as inhibitory cytokines (IL-10 and TGF-β1), checkpoint receptor ligands (PD-L1), cyclooxygenase-2 (COX-2) and death receptor ligands (FasL), which can ultimately impair T and NK-cell function (Whiteside, 2016).

### 2.6 Competitive metabolism of tumor cells

Tumor cells competitively metabolize and consume nutrients essential for effective immune cell function (Sukumar et al., 2015). For example, glucose is an important source of energy for HNSCC cell proliferation and survival as well as for immune cell activation, differentiation, and function. Tumor cell glycolytic activity may limit the glucose consumption of tumor infiltrating lymphocyte (TIL), resulting in T cell anergy and immune escape (Sandulache et al., 2011; Chang et al., 2015). Similarly, tryptophan is essential for T-lymphocyte growth and granzyme B production, and in the HNSCC TME, tumor cells can also catabolize tryptophan by releasing excess indoleamine-2,3-oxidase (IDO), ultimately impairing the immune response (Sukumar et al., 2017).

## 3 Application of immune checkpoint inhibitors in immunotherapy for HNSCC

**FIGURE 3:**
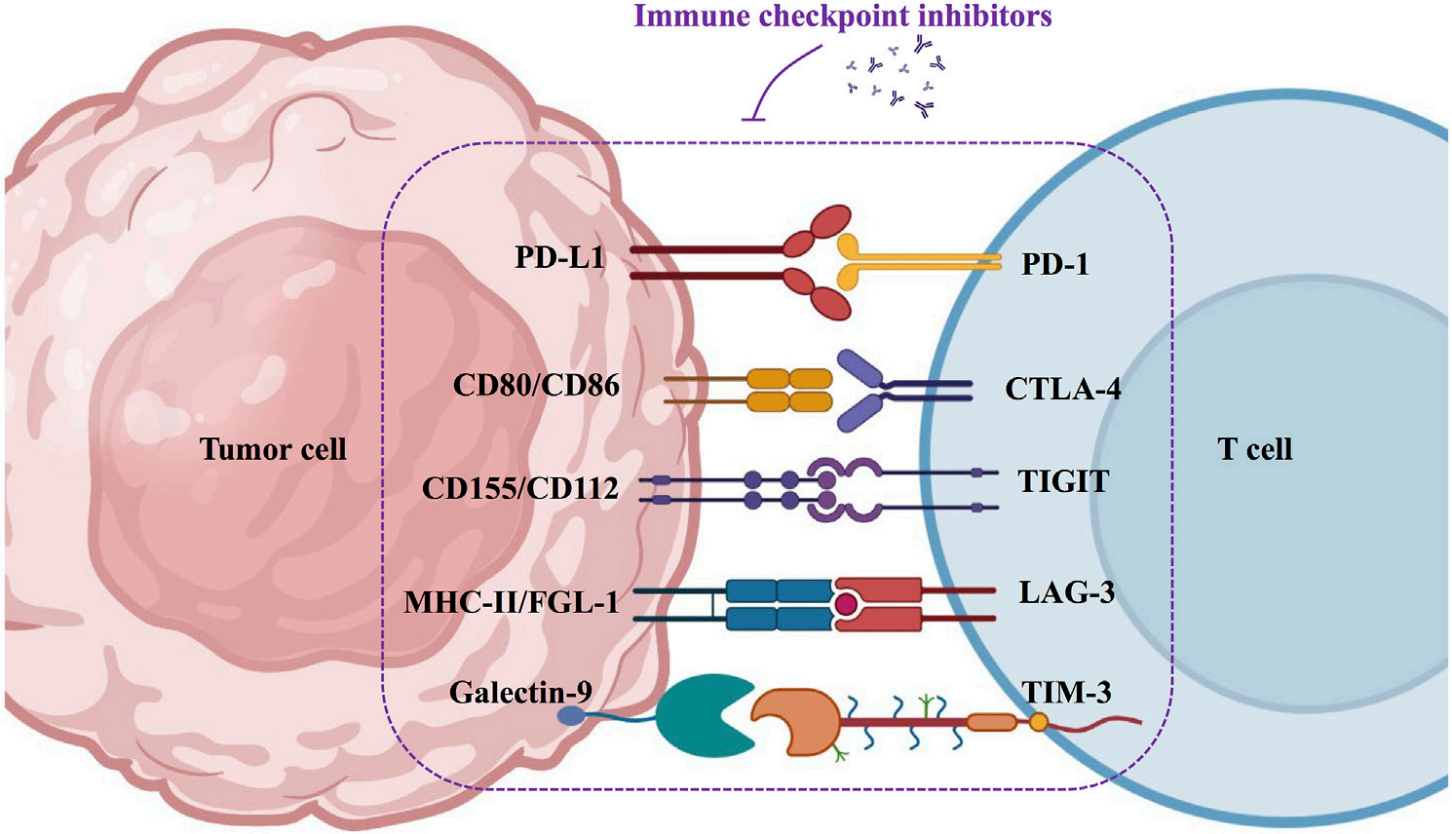
Schematic diagram of immune checkpoint inhibitors in immunotherapy for HNSCC.

In the past decades, dozens of studies and clinical trials have demonstrated the superiority of immunotherapy in prolonging the survival of patients with HNSCC. Immune checkpoint inhibitors have great prospects in immunotherapy, such as PD-1/PD-L1, CTLA-4, LAG-3, TIM-3, and TIGIT antibodies (Figure 3).

### 3.1 Programmed cell death protein-1 antibody

The interaction of PD-L1, which is highly expressed in tumor cells, and PD-1 on the surface of T cells inhibits the body’s immune response and promotes tumor progression and metastasis. By blocking the PD-1/PD-L1 pathway, the body’s antitumor immunity can be restored, and the effect of tumor immunotherapy can be achieved. Inhibitors of PD-1 that have been tested in HNSCC include pembrolizumab and nivolumab.

Pembrolizumab is an IgG4 monoclonal antibody. In the phase I trial KEYNOTE-012, pembrolizumab was 18% effective in the treatment of patients with recurrent metastatic HNSCC (Seiwert et al., 2016), and its safety and durable antitumor activity were confirmed by long-term follow-up (Mehra et al., 2018). Subsequently, in phase II KEYNOTE-055, pembrolizumab was shown to have meaningful antitumor activity and safety in recurrent and/or metastatic (R/M) HNSCC previously treated with platinum and cetuximab (Bauml et al., 2017). In the continuing phase III KEYNOTE-040 trial, pembrolizumab for R/M HNSCC demonstrated a stable safety profile accompanied by prolonged overall patient survival compared to the standard treatment arm (standard doses of methotrexate, doxorubicin, or cetuximab intravenously) (Cohen et al., 2019a). The phase III KEYNOTE-048 clinical trial opens a new era of first-line treatment for R/M HNSCC, and this trial measured the expression levels of PD-L1 to assess the effect of immunotherapy. The combined proportional score (CPS) is defined as the sum of PD-L1-stained tumor cells and surrounding lymphocytes and macrophages divided by the total number of live tumor cells multiplied by 100 (Cohen et al., 2019b). The results showed that pembrolizumab monotherapy improved survival in PD-L1-positive (CPS ≥ 1) patients compared to the extreme regimen, with a more significant benefit in PD-L1 strongly positive (CPS ≥ 20) patients (median overall survival, mOS: 14.9 vs. 10.8 months). Pembrolizumab in combination with chemotherapy prolonged mOS in the overall population, resulting in a survival of >4 years in nearly 1/5 patients (Burtness et al., 2019).

Nivolumab is another PD-1 monoclonal antibody approved for the second-line treatment of R/M HNSCC. In the phase III CHECKMATE-141 clinical trial, Ferris et al. divided 361 subjects into nivolumab and standard treatment groups (methotrexate, docetaxel, or cetuximab), with the nivolumab-treated group demonstrating a higher mOS (7.5 months vs. 5.1 months). Moreover, the probability of grade three or four adverse events was 13.1% in the nivolumab treatment group compared to 35.1% in the standard treatment group (Ferris et al., 2016). Immediately afterward, the team collected follow-up data for 24 months and concluded, consistent with the preliminary results, that nivolumab provided a longer-term, safer overall survival (OS) benefit in the treatment of R/M HNSCC, with an almost 3-fold increase in the 24-months OS rate compared to the standard treatment group (16.9% vs. 6.0%) (Ferris et al., 2018a). Further recent clinical trials on CheckMate 141 have also confirmed that nivolumab improves treatment outcomes regardless of prior treatment with cetuximab in R/M HNSCC patients and that the use of nivolumab results in a further reduction in the risk of death compared to single-agent chemotherapy (Ferris et al., 2019). In addition, nivolumab has also been reported to show a similarly good prognosis in Asian populations (Yen et al., 2020). Recently, a CheckMate 358 trial demonstrated that nivolumab induced pathological regression in HPV-positive and HPV-negative HNSCC (Ferris et al., 2021), and these results will provide a reference for investigating the addition of anti-PD-1 to adjuvant therapy in patients with locally advanced disease.

### 3.2 Programmed cell death protein-L1 antibody

PD-L1, one of the ligands of PD-1, is upregulated in HNSCC as well as in many types of solid tumors, and in recent studies, it has been shown that patients with high PD-L1 expression achieve better antitumor response and OS in immunotherapy (Patel et al., 2020). However, PD-L1 expression has been found to vary throughout the course of HNSCC, and patients with relapsed disease were advised to have their PD-L1 expression levels re-evaluated (Delafoy et al., 2022).

Durvalumab is a high-affinity humanized IgG1 monoclonal antibody targeting PD-L1 that blocks the binding of PD-L1 to PD-1 and CD80/B7.1, restores the immune response, and kills tumor cells (Chen et al., 2022). Durvalumab was used to evaluate the safety of treating R/M HNSCC in a phase I/II study with promising results (Segal et al., 2019). A phase II HAWK study (NCT02207530) was conducted in R/M HNSCC patients with PD-L1 expression ≥25% who had progressed after prior platinum-based chemotherapy. The subjects were treated with durvalumab monotherapy, which demonstrated good antitumor activity and safety (Zandberg et al., 2019). A higher objective response rate (ORR: 29.4% vs. 10.9%) and longer overall survival (OS: 10.2 vs. 5.0 months) were also observed with durvalumab in HPV-positive patients (Zandberg et al., 2019). Next, the Phase III EAGLE study (NCT02369874) (Ferris et al., 2020) and the Phase II CONDOR study (NCT02319044) (Siu et al., 2019) compared the effects of durvalumab monotherapy or in combination with the CTLA-4 inhibitor tremelimumab. The results showed that there was not a significant difference in OS; however, the higher survival and response rates at 12–24 months demonstrated the clinical efficacy of durvalumab.

Avelumab is another anti-PD-L1 antibody that has been explored in clinical trials in combination with chemoradiotherapy for the treatment of HNSCC. A phase I trial (NCT02938273) reported the feasibility of cetuximab-RT in combination with avelumab (Elbers et al., 2020). A phase Ib trial (NCT01772004) in which patients with platinum-refractory/ineligible R/M HNSCC were treated with avelumab, demonstrated controlled safety and clinical activity (Guigay et al., 2021). The safety phase of the randomized phase III trial (NCT02999087) then demonstrated the tolerability of the combination of avelumab + cetuximab-RT for LA-HNSCC and will advance into further clinical trials (Tao et al., 2020). One phase III clinical trial has been completed and published (NCT02952586). A total of 697 patients with locally advanced HNSCC were divided into an avelumab group (*n* = 350, avelumab + chemoradiotherapy) and a placebo group (*n* = 347, chemoradiotherapy) by Lee et al. (2021). The results showed that dismal asavelumab did not improve patients’ progression-free survival when compared to standard-of-care chemoradiotherapy alone. These data, however, provide a foundation for future research, and the authors predict that avelumab paired with chemoradiotherapy could provide significant benefits to patients with HNSCC who have high PD-L1 expression.

Atezolizumab has also been investigated in the treatment of HNSCC, with the combination of atezolizumab and GDC-0919 (IDO1 inhibitor) showing a controlled safety profile in a phase I clinical trial (NCT02471846) in advanced HNSCC (Jung et al., 2019). In addition, atezolizumab monotherapy (NCT03452137), atezolizumab in combination with radiotherapy (NCT05053737), atezolizumab in combination with VEGF-A-targeting monoclonal antibody bevacizumab (NCT03818061), and atezolizumab presurgery (NCT05110781) are being investigated for HNSCC (Garcia et al., 2020). The efficacy of the treatment deserves further observation in the future.

### 3.3 Cytotoxic T-lymphocyte-associated protein-4 antibody

CTLA-4, also known as CD152, is a CD28 homolog expressed on the surface of T-lymphocytes. CTLA-4 competes with CD28 for binding to the antigen-presenting cell (APC) surface ligands CD80 and CD86. CTLA-4 has a higher affinity for CD80/CD86 than CD28, resulting in dephosphorylation of T cell receptor (TCR) signaling proteins such as CD3, which mediates T cell unresponsiveness and thus participates in the negative regulation of the immune response (Rowshanravan et al., 2018). CTLA-4 inhibitors are currently being used in clinical trials primarily as combination therapies. Jie et al*.* demonstrated that ipilimumab contributes to the clinical efficacy of cetuximab treatment by targeting CTLA-4^+^ Tregs and restoring the effects of cetuximab-mediated antibody-dependent cell-mediated cytotoxicity (ADCC) and NK cells (Rowshanravan et al., 2018). A phase I clinical trial (NCT01935921) confirmed the safety of ipilimumab, and the recommended phase II dose (RP2D) for ipilimumab plus cetuximab-RT was 1 mg/kg in weeks 5, 8, 11, and 14 (Ferris et al., 2022). A phase II clinical trial (NCT02919683) failed to demonstrate that nivolumab in combination with ipilimumab was superior to nivolumab alone in untreated HNSCC (Schoenfeld et al., 2020). The phase III CheckMate-651 study evaluated the efficacy of nivolumab in combination with ipilimumab versus EXTREME in the first-line treatment of R/M HNSCC. Unfortunately, the effect of the immunotherapy combination did not provide a statistically significant improvement in OS (Argiris et al., 2021). Current clinical studies on tremelimumab have focused on its combination with durvalumab in R/M HNSCC, but as previously mentioned (Siu et al., 2019; Ferris et al., 2020), the application of tremelimumab did not significantly benefit HNSCC patients.

### 3.4 Lymphocyte activation gene 3 protein antibody

LAG-3 (CD223) is another helper suppressor checkpoint molecule in HNSCC that is expressed primarily on activated T cells and, to a lesser extent, also on NK cells, DCs, and B cells.

LAG-3 inhibits T-cell activation by competing with CD4 for binding to major histocompatibility complex class II (MHC-II) (Huard et al., 1997). In addition, LAG-3 is highly expressed on Tregs of HNSCC patients (Jie et al., 2013). Recent evidence suggests that LAG-3 exhibits significant upregulation in HPV-associated HNSCC compared to HPV-independent HNSCC. Interestingly, this observation was not detected in other pathways, such as PD-1 and CTLA-4, and appears to be specific to LAG-3, suggesting that HPV-associated HNSCC may benefit significantly from LAG-3 blockade (Panda et al., 2020). The current rationale for LAG-3 inhibitors is entirely centered on blocking the LAG-3/MHC-II pathway. Notably, Wang et al. introduced another ligand of LAG-3, Fibrinogen-like protein 1 (FGL1), and suggested a potential new target in cancer immunotherapy, the FGL1/LAG-3 pathway (Wang et al., 2019). Blockade of the FGL1-LAG-3 interaction by monoclonal antibodies stimulates tumor immunity in a receptor–ligand interdependent manner.

Deng et al. (2016) established a preclinical model of HNSCC mice, which ultimately validated the efficacy of LAG-3 blockade *in vivo*. By blocking LAG-3 through the infusion of anti-mouse LAG-3 antibodies, the investigators observed an inhibition of tumor progression in mice, along with a significant increase in the antitumor response mediated by CD8^+^ T cells and a consequent reduction in the number of immunosuppressive cells, such as Tregs, and MDSCs, in the HNSCC mouse model. In addition, preclinical studies by Mishra et al. (2016) also suggested that there may be synergistic effects between LAG-3 and the PD-1/PD-L1 pathway and that dual blockade of PD-1 and LAG-3 may enhance the antitumor effects. To date, LAG-3 inhibitors have been tested in phase I/IIa studies in patients with recurrent melanoma, and effective results have been obtained (Atkinson et al., 2020; Tawbi et al., 2022). Eftilagimod (IMP321) and relatlimab (BMS-986016) are currently available inhibitors against LAG-3. Clinical studies of eftilagimod for HNSCC are underway. The TACTI-002 study combines eftilagimod with pembrolizumab in R/M HNSCC to assess safety and efficacy, with objective response rate (ORR) as the primary outcome indicator, and is expected to be completed in May 2023. Two clinical studies on relatlimab are also ongoing. NCT04080804 will treat locally advanced HNSCC with relatlimab adjuvant to nivolumab. Another phase II study, NCT04326257, randomized patients to either the nivolumab + relatlimab or nivolumab + ipilimunmab arm to assess the ORR of both treatment regimens in patients with R/M HNSCC. It is expected that the addition of LAG-3 inhibitors will bring further survival benefits to HNSCC patients.

### 3.5 T cell immunoglobulin and ITIM domain antibody

TIGIT exhibits high expression on tumor-infiltrating lymphocytes (TILs) and mediates the suppression of effector T cells and NK cells upon binding to their ligands CD155 and CD112 (Harjunpää and Guillerey, 2020). The TIGIT inhibitor tiragolumab is currently being used primarily in combination with anti-PD-L1 drugs in clinical trials in HNSCC, with two phase II studies (NCT03708224 and NCT04665843) underway. The former will determine the effect of tiragolumab in combination with atezolizumab on T-cell infiltration in advanced HNSCC. The latter will assess the safety and therapeutic efficacy of tiragolumab and atezolizumab in the treatment of R/M PD-L1-positive HNSCC. In addition, phase I clinical trials of various novel anti-TIGIT humanized monoclonal antibodies in combination with anti-PD-1 agents for the treatment of advanced solid tumors have been conducted, including MK-7684 in combination with pembrolizumab (NCT05007106), ASP8374 in combination with pembrolizumab (NCT03260322) and BMS-986207 in combination with nivolumab (NCT02913313).

### 3.6 Mucin domain-3 antibody

TIM-3 was shown to be coexpressed with PD-1 on the surface of TILs. A preclinical study by Shayan et al. (2017) observed compensatory upregulation of TIM-3 expression in a murine HNSCC model treated with anti-PD-1. Moreover, Jie et al. (2017) reported that simultaneous blockade of TIM-3 and PD-1 during cetuximab treatment may improve the survival benefit of HNSCC patients. A phase Ia/Ib study presented at the 2019 ASCO-SITC Clinical Immuno-Oncology Symposium reported good tolerability of anti-TIM-3 antibody (LY3321367) monotherapy or in combination with anti-PD-1 antibody (LY3300054) in advanced solid tumors, including HNSCC (Harding et al., 2019). A phase I dose-escalation study (NCT03708328) is ongoing to evaluate the safety and preliminary antitumor activity of a PD-1/TIM-3 bispecific antibody (RO7121661) in patients with advanced or metastatic solid tumors, including HNSCC.

## 4 Costimulatory agonists for HNSCC

**FIGURE 4:**
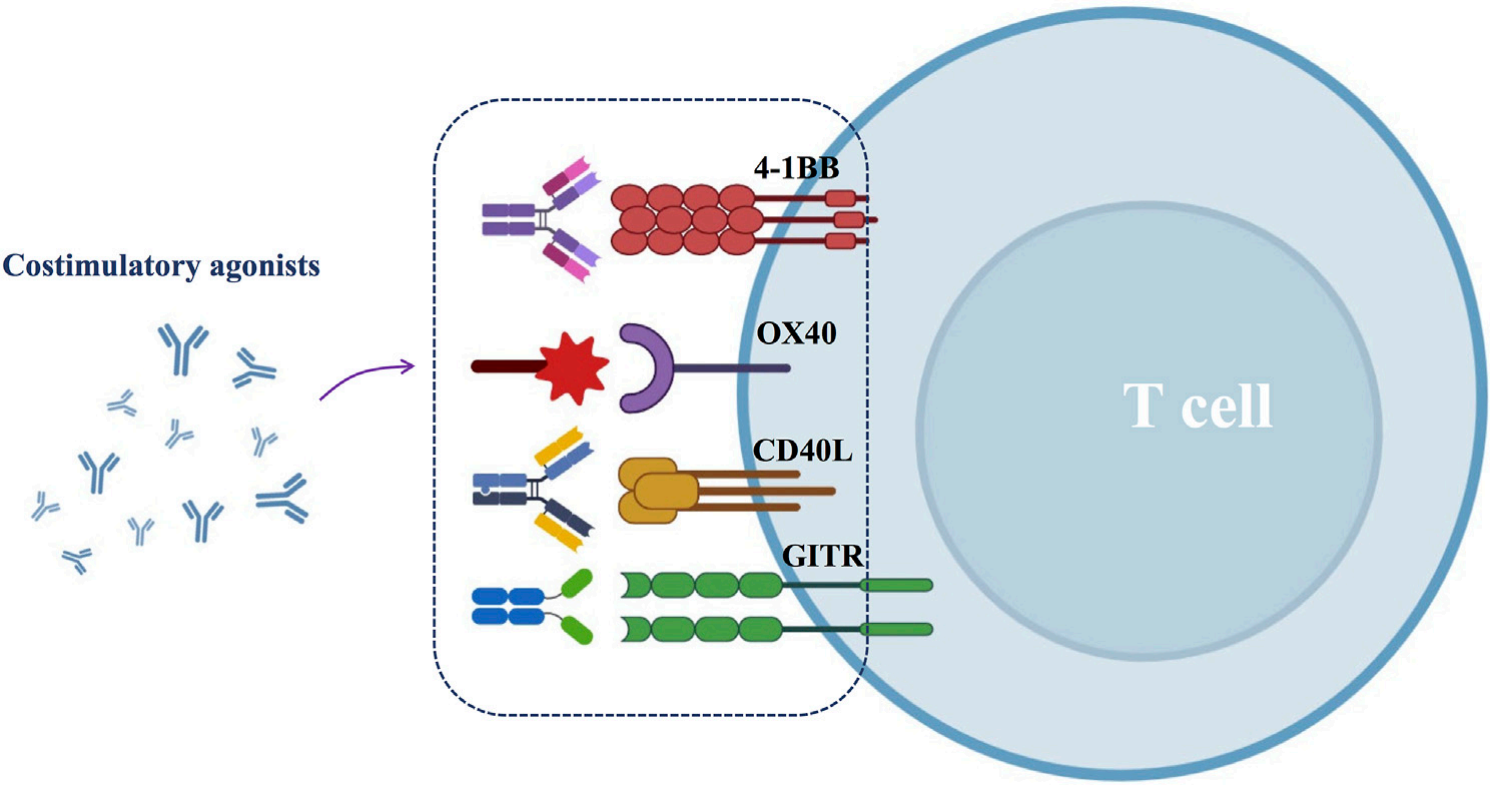
Schematic diagram of costimulatory agonists for HNSCC.

Despite the efficacy of ICBs, most HNSCC patients still develop progressive disease necessitating additional treatment options. One approach is the application of costimulatory agonists, which promote T-cell activation, and the generation of long-lived memory T cells. Common costimulatory agonists include 4-1BB, OX40, CD40 and GITR agonists (Figure 4).

### 4.1 4-1BB (CD137) agonist

4-1BB (CD137) is a member of the tumor necrosis factor receptor (TNFR) family, a costimulatory receptor expressed on activated T cells, NK cells, and DC cells (Liao et al., 2019). Activated by its ligand 4-1BBL, 4-1BB promotes the differentiation of effector T cells and suppresses the function of Tregs. In addition, activated 4-1BB can coordinate IL-15 and IL-21 to drive NK-cell proliferation and enhance ADCC effects, thus exerting antitumor immune effects (Vidard et al., 2019). Vahle et al. (2016) developed a murine head and neck tumor model and confirmed the antitumor effects of CD137 agonists by performing fluorodeoxyglucose (FDG)-positron emission tomography (PET)-CT and FDG-PET-MRI assessments. Another study demonstrated that the use of CD137 agonists in HNSCC patients promoted cetuximab-induced DC maturation and enhanced the cross-presentation of HNSCC antigens by DCs (Srivastava et al., 2017). Urelumab (BMS-663513) and utomilumab (PF-05082566) are two immune-activating antibodies against the 4-1BB target. The use of urelumab alone has been shown to cause severe hepatotoxicity, thought to be associated with doses of >1 mg/kg (Segal et al., 2017). However, urelumab may enhance the efficacy of other monoclonal antibodies in patients with advanced HNSCC, such as cetuximab (Srivastava et al., 2017). Furthermore, a phase Ib study (NCT02179918) confirmed the safety and clinical activity of utomilumab in combination with pembrolizumab in the treatment of patients with advanced solid tumors, including HNSCC (Tolcher et al., 2017). Therefore, it can be speculated that when a 4-1BB agonist is used in combination with other monoclonal antibodies, it may have an enhanced antitumor effect in the treatment of HNSCC. Additionally, hepatotoxicity could be attenuated by reducing the 4-1BB agonist dose. More clinical studies are still needed to support this hypothesis.

### 4.2 OX40 (CD134) agonist

OX40 (CD134) is one of the TNFRs and is present on T cells, especially CD4^+^ T cells. When activated with its ligand OX40L, OX40 enhances effector T cell-mediated killing of tumor cells (Loick et al., 2022). OX40 levels on the surface of CD4^+^ T cells were found to be significantly lower in patients with HNSCC and more pronounced in advanced HNSCC (Baruah et al., 2012). OX40 target-related drugs for the treatment of HNSCC are still in early clinical trials. MEDI0562, an agonist of OX40, was safely administered to patients with advanced solid tumors (47% HNSCC) in a phase I dose-escalation study (NCT02318394) at doses up to 10 mg/kg (Glisson et al., 2020). Another phase Ib clinical trial (NCT02274155) tested a murine anti-human OX40 agonist antibody (MEDI6469). In this trial, 17 patients with locally advanced HNSCC received MEDI6469 prior to surgery, and the results showed that the application of OX40 agonist increased the activity of CD4^+^ and CD8^+^ T cells in blood and tumors, while its safety was confirmed (Duhen et al., 2021). In addition, it has been demonstrated that the antitumor activity of OX40 agonist therapy is further enhanced when combined with anti-PD-1, anti-CTLA-4, cytokines, chemotherapy, or radiotherapy (Fu et al., 2020). These results provide a new combination treatment option for patients with HNSCC, and further clinical trials are still required to confirm this strategy.

### 4.3 CD40 agonist

CD40 is a costimulatory receptor molecule expressed on the surface of antigen presenting cells (APCs), monocytes, and tumor cells. It exerts immunomodulatory effects through binding to its ligand CD40-L (CD154). Sathawane et al. (2013) found that CD40 expression gradually decreased as HNSCC progressed from stage I to IV and was elevated again when the tumor was surgically resected. This finding suggests that the expression level of CD40 may be regulated by tumor load. A recent study investigated a murine model of homologous tongue cancer. The tumor was resected by partial glossectomy, and CD40 agonist and/or PD-1 antagonist therapy was administered postoperatively. Ultimately, the increased survival rate in mice suggests that the combination of the two is probably an effective postoperative adjuvant therapy (Ahn et al., 2020). Labiano et al. (2021) developed a novel fibroblast activation protein (FAP)-targeted CD40 agonist (FAP-CD40). In this work, the investigators established a homologous HPV-HNSCC murine model. They treated tumor-bearing mice with local hypofractionated radiotherapy (2 × 6 Gy) alone or in combination with a systemic administration of the FAP-CD40 antibody. The results showed that combination therapy induces durable and effective responses in 80% of tumor-bearing mice. Another study by Monteiro de Oliveira Novaes et al. (2021) reported that in a carcinogen-induced murine model of OSCC, activation of the CD40 pathway, as well as PD-1/PD-L1 pathway blockade, was able to prevent oral premalignant lesion (OPL) progression into invasive OSCC. CD40 agonist antibodies currently have limited application in the treatment of HNSCC due to the side effects exhibited by systemic administration (Stone et al., 2021). In the latest phase I study (NCT02955251), Jason *et al.* evaluated ABBV-428, a mesothelin-CD40 bispecific antibody that interacts with the tumor antigen mesothelin, and achieved CD40 activation in the tumor microenvironment, resulting in localized, nonsystemic immune activation. In this study, 59 patients with advanced solid tumors, including HNSCC, were treated at doses ranging from 0.01 to 3.60 mg/kg. The results showed that ABBV-428 monotherapy exerted an acceptable safety profile, confirming that tumor-targeting bispecific antibodies can improve the safety of CD40 agonists as a therapeutic approach (Luke et al., 2021).

### 4.4 GITR agonist

Glucocorticoid-induced TNFR family-related gene (GITR) is a new costimulatory molecule whose activation reduces the recruitment of Tregs in the HNSCC TME and increases T cell proliferation, thereby enhancing the antitumor effect (Buzzatti et al., 2020). A preclinical animal model trial found that simultaneous targeting of PD-1 and GITR synergistically enhanced CD8^+^ T cell memory and antitumor function, and these encouraging results may provide a new direction for the clinical treatment of HNSCC (Wang et al., 2018). A phase I study (NCT02437916) of AMG228, a stimulatory human IgG1 monoclonal antibody conjugated to GITR, was completed in 2018. Thirty patients with advanced solid tumors (including 10 with HNSCC) were treated with AMG228 monotherapy. AMG228 demonstrated a good safety profile. Unfortunately, the investigators were unable to observe reliable clinical effects and antitumor activity in the trial and therefore were unable to initiate a phase two dose expansion study (Tran et al., 2018). Clinical trials are ongoing in the investigation of INCAGN01876 (another GITR agonist) in the treatment of HNSCC. A completed phase I/II clinical study (NCT03126110) combined INCAGN01876, nivolumab, and ipilimumab in advanced or metastatic malignancies, including HNSCC. Unfortunately, there were no positive results to demonstrate the efficacy of INCAGN01876. An ongoing clinical trial (NCT03088059) will treat R/M HNSCC with INCAGN01876 in combination with anti-PD-1/anti-PD-L1 antibody. In addition, INCAGN01876 is also being used in combination in a phase Ib study (NCT04470024) of a multivalent autophagosomal vaccine (DPV-001) for the treatment of R/M HNSCC. The outcomes of these clinical trials remain to be seen.

## 5 Antigenic vaccines for HNSCC

In cancer cells, many neoantigens are produced and expressed by nonsynonymous gene mutations. Their lack of expression in normal tissues and strong immunogenicity make these neoantigens ideal targets for immunotherapy (Peng et al., 2019). Antigenic vaccines are designed to activate the immune response by increasing antigen-specific CD4^+^ and CD8^+^ T cells after vaccination, ultimately mediating the regression of tumor cells.

### 5.1 HPV-related antigenic vaccines for HNSCC

The incidence of HPV-associated HNSCC has increased dramatically over the last two decades, and HPV-associated oncoproteins such as E6 and E7 are ideal targets for therapeutic vaccines due to their consistency and uniqueness (Gillison et al., 2000; Tan et al., 2018). HPV-related antigenic vaccines are available in many forms, such as peptide and DNA vaccines.

#### 5.1.1 HPV-related peptide-based vaccines

Peptide-based vaccines are composed of amino acid sequences containing epitopes that cause the immune system to respond. When patients are vaccinated with synthetic tumor-specific or tumor-associated peptides or combinations of peptides, these peptides are presented on human leukocyte antigen (HLA) molecules on the cell surface, inducing and activating CD4^+^ and CD8^+^ T cells, resulting in powerful therapeutic effects.

In an earlier phase I study, Dan *et al.* used a panel of peptide immunomodulatory vaccines GL-0810 (targeting HPV16 E7) and GL-0817 (targeting the melanoma-associated antigen A3, MAGE-A3) to treat HPV16- and MAGE-A3-positive R/M HNSCC patients. They were split into two groups, with nine on GL-0810 and seven on GL-0817. Ultimately, 80% of the HPV16 E7 cohort and 67% of the MAGE-A3 cohort developed a significant antibody response to the vaccine. Moreover, in patients with R/M HNSCC, GL-0810 and GL-0817 were found to be well tolerated (Zandberg et al., 2015). Researchers also recently evaluated the role of an optimized nanoparticle-conjugated E7 long-peptide vaccine (NP-E7LP) in a mouse model of HPV-associated HNSCC. Fifteen mice were vaccinated with NP-E7LP prior to surgical resection of primary tumors. The results showed that NP-E7LP-vaccinated mice had no postsurgical tumor recurrence (0/15), whereas the CpG and PBS controls had a high recurrence rate (3/13 and 5/8, respectively). The researchers concluded that combining vaccination with tumor resection exposed residual tumor cells to preexisting vaccine-induced T cells, preventing local tumor recurrence. Patients with HPV-associated HNSCC may expect to benefit from future clinical trials combining E7 vaccination and surgical resection (Domingos-Pereira et al., 2021).

Next, a multipeptide vaccine targeting both HPV16 E6 and/or E7 was tested. Multipeptide vaccines are expected to provide better disease control by preventing antigen loss and lowering the risk of immune escape. PDS0101 is a therapeutic peptide vaccine against HPV16 E6/E7. Claire *et al.* reported that monotherapy with PDS0101 produced HPV-specific T cells and antitumor activity in mice bearing HPV-expressing mEER oropharyngeal carcinoma. Additionally, maximum antitumor effects were achieved when PDS0101 was combined with bintrafusp alfa, a bifunctional fusion protein targeting TGF-β and PD-L1, and NHS-IL12, an immune cytokine targeting tumors and designed to deliver IL-12 to the TME (Smalley Rumfield et al., 2020). A recent phase I/II study (NCT04287868) further expanded the use of PDS0101 by testing a triple combination therapy of PDS0101, bintrafusp alfa, and M9241 (a tumor-targeting immunocytokine composed of IL-12 heterodimers fused to a monoclonal antibody targeting free DNA in necrotic tumor regions). Interim results of the trial were reported at the 2021 ASCO meeting: 14 patients with advanced HPV-positive cancer were enrolled in the study (including 3 cases of oropharyngeal cancer), and of these, 5/6 (83%) patients with checkpoint-insensitive disease and 5/8 (63%) patients with checkpoint-refractory disease had objective responses. Further analysis of the clinical immune response is ongoing, and more positive results are expected (Strauss et al., 2021).

In addition, there are peptide vaccines targeting HPV-associated TAAs. The cyclin-dependent kinase inhibitor p16 (INK4a) is upregulated in all HPV-associated cancers. In a phase I/IIa study by Miriam *et al.*, 24 patients with advanced HPV-associated cancer, including 6 with HNSCC, received vaccination with the p16(INK4a)-derived peptide P16_37-63. Fourteen of 20 patients had specific CD4^+^ T cells, five of 20 patients had specific CD8^+^ T cells, and 14 of 20 patients had antibodies directed against the targeted protein. The results demonstrated that the p16 (INK4a) peptide vaccine induced cellular and humoral immune responses and had an acceptable safety profile (Reuschenbach et al., 2016).

#### 5.1.2 HPV-related DNA vaccines

DNA vaccines are made from bacterial plasmids that can integrate multiple genes to encode multiple tumor antigens, allowing for more precise and efficient immune responses.

MEDI0457 is a DNA vaccine that targets HPV16/18 E6/E7 with IL 12-encoding plasmids. Charu *et al.* reported positive results in a phase Ib/II clinical trial involving 22 patients with HPV-associated locally advanced HNSCC who received MEDI0457, with 18 of the 21 evaluable patients harboring elevated levels of antigen-specific T cells. All five of the post immunotherapy tumor samples had more perforin-expressing immune infiltrates, with four of them having higher CD8/Foxp3 ratios (Aggarwal et al., 2019). Another phase Ib/IIa study (NCT03162224) examined the safety and efficacy of MEDI0457 in combination with anti-PD-L1 durvalumab for HPV^+^ R/M HNSCC. The results showed an ORR of 22.2% with three CRs and three PRs. There was also an increase in tumor-infiltrating CD8^+^ T cells and HPV-specific T cells in the peripheral blood (Aggarwal et al., 2020). In addition, Chandra et al. (2021) have developed an alternative therapeutic HPV DNA vaccine (AMV002). In the phase I dose-escalation study (ACTRN12618000140257), AMV002 was used to assess the tolerability and immunogenicity in the treatment of HPV-associated HNSCC. The escalating AMV002 dose was administered intradermally (ID) to the forearm (from 0.25 mg/dose to 4 mg/dose). The final 12 subjects showed good tolerability at all dose levels, and 83.3% displayed HPV16 E6/E7-specific T cell immune responses after vaccination.

Overall, the use of therapeutic vaccines in HPV-associated HNSCC is currently limited to animal studies and phase I/II clinical trials. Both peptide and DNA vaccines have been shown to have promising application potential. However, no relevant phase III studies are currently available.

### 5.2 DC-based vaccines

**FIGURE 5:**
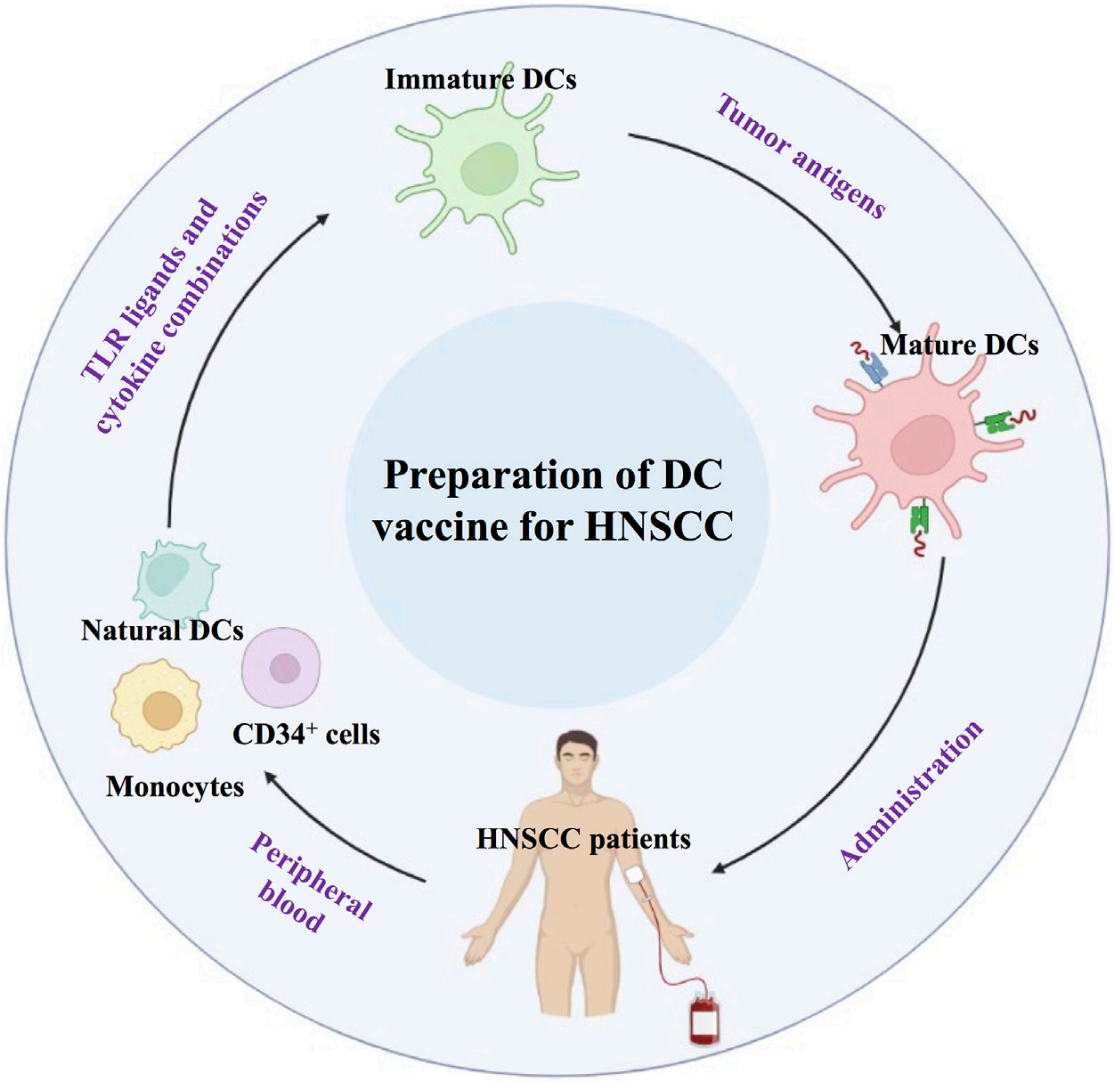
Schematic diagram of applying DC-based vaccination in HNSCC.

Dendritic cells (DCs) are the most important APCs, and DC-based vaccines play an important role in antitumor biotherapy (Dong et al., 2018a; Liu et al., 2018; Chen et al., 2019). The safety of DC-based immunotherapy has been well documented in many phase I and II clinical trials. The side effects seen with the majority of DC vaccination protocols were minimal and self-limiting. Current approaches for DC-based vaccination rely primarily on antigen loading on *in vitro*-generated DCs derived from monocytes or CD34^+^ cells, activating them with various TLR ligands and cytokine combinations (Dong et al., 2018b). Activated DCs could be injected back into HNSCC patients to promote a cytotoxic T cell response (Figure 5).

DC vaccination in HNSCC patients aims to induce or augment an effective antitumor immune response against HNSCC tumor antigens. *Ex vivo* culture of DCs involves natural DCs from precursor cells, which are obtained from peripheral blood. Immature DCs can be obtained and induced to mature DCs. Subsequently, mDCs are administered to HNSCC patients.

TP53 encodes p53, which is the most common genomically altered gene in HPV-negative tumors and a valuable target for HPV-negative HNSCC vaccines (Chung et al., 2015). A vaccine based on autologous monocyte-derived DCs loaded with selected wild-type p53 peptides was reported by Patrick *et al.* in a phase I clinical trial (NCT00798655) published in 2014 (Schuler et al., 2014). In this trial, 69% of HNSCC patients (11 out of 16) had increased levels of p53-specific T cells after vaccination. In addition, Treg levels continued to decrease after vaccination in all patients. The two-year disease-free survival rate was 88%. Apart from this, no II-IV adverse events were observed in any of the subjects. The results demonstrated the feasibility of a p53-specific adjuvant vaccine in the treatment of HNSCC. The authors also suggested that DCs in HNSCC patients require stronger maturation stimuli to reverse immunosuppression and improve vaccine efficacy (Schuler et al., 2014).

In 2016, Theresa *et al.* described a DC vaccine made from DCs derived from monocytes loaded with apoptotic tumor cells (ATCs). Although the study was suspended because not enough subjects were recruited, it demonstrated potential safety and immunogenicity in all four subjects receiving the vaccination for stage III/IV HNSCC. Together with the low sensitivity of the DC vaccine production technique, this provides an optimistic direction for the development of a clinical HNSCC vaccine (Whiteside et al., 2016). A recent I/II pilot study (UMIN 000027279) described another vaccination with Wilms’ tumor 1 (WT1) peptide-loaded DCs. When combined with conventional chemotherapy in 11 patients with R/M HNSCC, no serious adverse events associated with vaccination were observed. The median progression-free survival and overall survival were 6.4 and 12.1 months, respectively. It was also observed that DC vaccination enhanced WT1-specific immunity (Ogasawara et al., 2019).

### 5.3 Tumor-associated antigen vaccines

Several vaccines based on different tumor antigens have been developed. The first is cancer vaccines against TAAs, a type of vaccine that provides off-the-shelf treatment for most patients with HPV-negative HNSCC. The most recent study involved DNA-based vaccines (pDom-M/F) targeting MAGED4B and FJX1 (two TAAs), which were expressed at higher levels in HNSCC tumor samples than in normal tissue. Additionally, MAGED4B- and FJX1-specific T cells can be detected at high frequencies in HNSCC patients. pDom-M/F is made from full-length sequences of MAGED4B or FJX1 linked to 3′Dom sequences and inserted into pcDNA3 plasmids. The results in preclinical models were promising, with inoculation of pDom-M/F increasing T-cell infiltration and mediating delayed tumor growth in murine tumor models. Furthermore, when prom-M/F was combined with anti-PD-1 treatment, it demonstrated excellent antitumor effects, with complete tumor suppression achieved in six (75%) of the eight mice (75%) treated with the combination (Wang et al., 2021c).

Germline antigens are another target of TAAs. In an early phase II clinical trial (CTR-8379), Yoshihiro *et al.* innovatively used peptides derived from cancerous testicular antigens, including lymphocyte antigen six complex locus K (LY6K), cell division cycle associated gene 1 (CDCA1), and insulin-like growth factor-II mRNA-binding protein 3 (IMP3), to synthesize a peptide vaccine for the treatment of patients with advanced HNSCC. Based on good tolerability, 37 vaccinated subjects demonstrated significantly longer median survival time than 18 negative controls (MST: 4.9 months vs. 3.5 months). In the vaccination group, LY6K-, CDCA1-and IMP3-specific CTL responses were identified in 85.7%, 64.3%, and 42.9% of patients, respectively. At the same time, patients exhibiting a CTL response against three and two peptides demonstrated an extended OS (Yoshitake et al., 2015).

There have also been advances in vaccines targeting other TSAs. Mucin 1 (MUC1) is overexpressed in most T2-T3 HNSCCs and lacks complete glycosylation in HNSCC compared to normal tissue (Gao et al., 2020; Liao et al., 2021), making it another promising target for HNSCC. MUC1 is another promising target for HNSCC. In an interim analysis of a phase I trial (NCT02544880), Donald *et al.* reported an MUC1 vaccine with poly ICLC as an adjuvant. In combination with tadalafil, a PDE5 inhibitor, it was shown to be well tolerated in patients with recurrent HNSCC. Moreover, tadalafil and the MUC1 vaccine reduced both MDSCs and Tregs in peripheral blood and tumor sites, reversing immune rejection (Weed et al., 2019).

### 5.4 Personalized antigen vaccines

A recent animal study investigated a class of tumor membrane-based vaccines using tumors grown subcutaneously in mice to prepare tumor membrane vesicles (TMVs). These TMVs bound to immunoaffinity-purified mouse GPI-B7-1 and GPI-IL-12 molecules (GPI-ISMs) *via* protein transfer to produce the vaccine. In a murine model of HNSCC, the TMV vaccine induced an antitumor immune-memory response and showed antitumor synergy in combination with an anti-PD-1 monoclonal antibody. The findings suggest the prospect of a personalized therapeutic vaccine for HNSCC. The application of surgically removed tumor tissue to prepare TMV for the production of a personalized vaccine for individual patients in future clinical practice (Bommireddy et al., 2020).

At the recent 2021 ASCO meeting, MVX-ONCO-1, a new personalized vaccine, was announced. This vaccine is made from irradiated autologous tumor cells combined with genetically engineered allogeneic cells. In two clinical trials (NCT02193503 and NCT02999646), MVX-ONCO-1 was used to evaluate the treatment of 11 patients with locally advanced/metastatic HNSCC who had relapsed after receiving first-line systemic therapy. All patients received at least five doses of MVX-ONCO-1 over 8 weeks. Ten patients who had been followed up for at least 6 months were analyzed. All experienced no treatment-related adverse events (AEs) greater than grade two. On this basis, eight (80%) patients experienced varying degrees of tumor control, including four with stable disease (SD), two with partial response (PR), and two with complete response (CR), and the two patients who demonstrated CR did not continue with anticancer therapy for 24 and 6 months. The final results are exciting, and the clinical trial of MVX-ONCO-1 reveals the future promise of personalized vaccines in the treatment of HNSCC (Fernandez et al., 2021).

## 6 Oncolytic viral therapy

**FIGURE 6:**
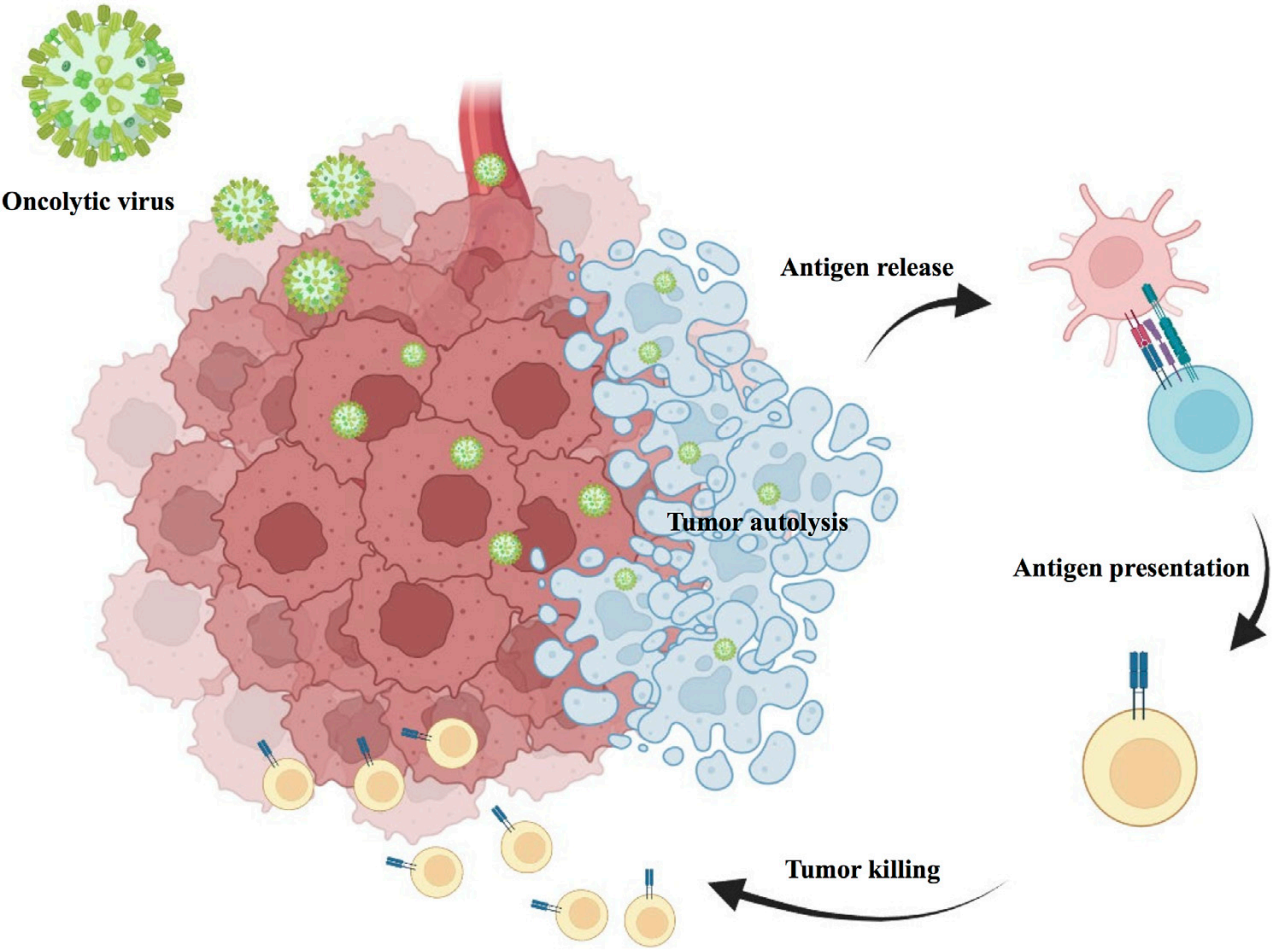
Immunomodulatory mechanisms of oncolytic viral therapy.

Oncolytic viral therapy is a revolutionary new therapy that uses attenuated strains of various viruses to directly kill tumor cells while inducing antitumor immune effects in the body (Goradel et al., 2021). Oncolytic viruses have a multimodal mechanism of action with both direct and indirect toxic effects on tumor cells, including autolysis, immune cell homing, destruction of vascular supply and potentiation of other adjunctive anticancer therapies (Figure 6). Talimogene laherparepvec (T-VEC) is a herpes simplex virus (HSV)-based oncolytic virus that was approved for clinical use by the US Food and Drug Administration (FDA) in 2015 after showing promising therapeutic results in phase I, II, and III clinical trials for the treatment of advanced melanoma (Andtbacka et al., 2015). A recent Ib study (NCT02626000) in R/M HNSCC used T-VEC in combination with pembrolizumab in 36 patients with R/M HNSCC, and the combination treatment was safely tolerated in the subjects. However, unfortunately, the addition of T-VEC did not improve efficacy compared to pembrolizumab monotherapy (Harrington et al., 2020).

Intratumoral inoculation of an oncolytic virus with transfection, oncolytic virus induced tumor autolysis, direct cell lysis of HNSCC tumor and immune cell recruitment, then result in tumor killing. Oncorine (H101) is an adenovirus-based tumor lysis virus. In an earlier phase III clinical trial by Xia et al. (2004), H101 intratumor injection was shown to be safe and effective in patients with HNSCC. Recent studies have revealed high expression of the oncogene SNHG1 in OSCC. More importantly, Wang et al. (2020) also found that H101 could exert better antitumor effects in OSCC with high SNHG1 expression. Onyx-015 is an adenovirus with the E1B (55 kDa) gene deleted. Several phase II clinical trials involving Onyx-015 in the therapy of HNSCC have yielded promising results (Lamont et al., 2000; Nemunaitis et al., 2000; Nemunaitis et al., 2001). However, phase III trials are still needed for confirmation. Lysozyme measles virus is also a potential treatment for HNSCC. A phase I trial (NCT01846091), currently underway, is designed to evaluate the safety and immunological effects of MV-NIS (an oncolytic measles virus encoding thyroidal sodium iodide symporter) in R/M HNSCC. Other naturally occurring lytic viruses, such as Newcastle disease virus (NDV), have been shown to have a potent lytic effect on HNSCC in early studies. However, further confirmations in clinical trials are needed (Li et al., 2011).

The current application of oncolytic viruses in the treatment of HNSCC faces two challenges. The first is to explore optimal treatment regimens for immunotherapeutic agents used in combination with oncolytic viruses. The second is to improve the delivery of the virus to the tumor and to mediate the antitumor effects precisely and effectively (Mondal et al., 2020).

## 7 Adoptive T cell therapy

**FIGURE 7:**
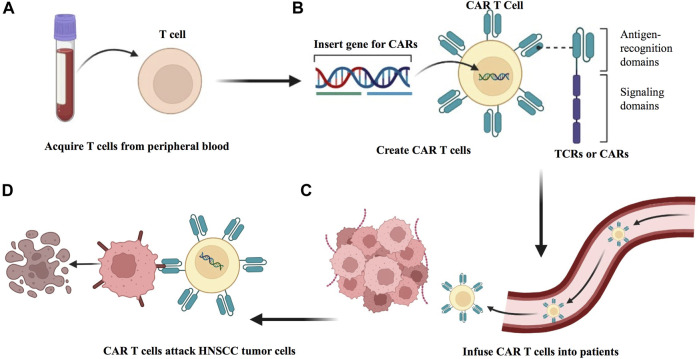
Preparation of ACT to treat HNSCC. **(A)** Adoptive transfer of antitumor T cells isolated from within the HNSCC tumor of the patient. **(B)** Tumor-infiltrating T cells (TILs) are extracted from surgically resected tumor samples and then expanded *in vitro*, followed by reinfusion into HNSCC patients. **(C)** T cells from patient peripheral blood are isolated and expanded in culture and genetically modified to express either a T cell receptor (TCR) or a chimeric antigen receptor (CAR). **(D)** The modified T cell confers the ability to specifically recognize and killing HNSCC tumor cells.

Adoptive T cell therapy (ACT) represents a personalized oncology treatment option. The principle is to exploit the specificity and antitumor effects of T cells by expanding tumor-specific T cells *in vitro* and finally transfusing them back to the patient to kill the tumor cells (Figure 7). To precisely target antigens expressed on tumors without damaging normal tissue cells, endogenous tumor-infiltrating lymphocytes (TILs) can be obtained and expanded from autologous tumor resection specimens or biopsies, or peripheral blood T cells can be genetically engineered *in vitro* with antitumor T cell receptors (TCRs) or chimeric antigen receptors (CARs) (Rosenberg and Restifo, 2015). ACT with TIL has been shown to exhibit curative cancer regression in metastatic melanoma (Rosenberg and Restifo, 2015). The application of ACT in HNSCC has also progressed.

An early study applied ACT to a murine HNSCC model and observed that ACT synergistically enhanced IL-2 cytokine gene therapy and mediated tumor regression (Ambade and Mulherkar, 2008). Another study analyzed 43 patients with HNSCC who received chemotherapy after radical tumor surgery, 21 of whom were included in the experimental group treated with ACT achieved a higher survival benefit (Jiang et al., 2015). Wei et al. (2021) isolated and cultured TILs from fresh tumor tissue of eight HNSCC patients and demonstrated the feasibility of isolating neoantigen-specific T cells from TILs. Moreover, researchers determined their TCR sequences and constructed TCR-engineered T cells, which eventually confirmed their protumor regression ability in a mouse model. Several clinical studies related to HNSCC are currently ongoing. NCT03083873 is evaluating the efficacy of autologous TILs (LN-145/LN-145-S1) in R/M HNSCC. NCT03645928, another phase II trial, is still in the recruitment phase aimed at treating patients with solid tumors, including HNSCC, with autologous TIL [LN144 (Lifileucel)/LN-145/LN-145-S1] in combination with or without a checkpoint inhibitor. Research related to TCR-engineered T cells in HNSCC is also in progress (NCT03247309). In addition, three clinical trials, NCT05117138, NCT03740256, and NCT05239143, will evaluate the safety and efficacy of AMT-116 CAR-T cells, HER2 chimeric antigen receptor-specific cytotoxic T lymphocytes (HER2-specific CAR T cells), and P-MUC1C-ALLO1 CAR-T cells, respectively, in the treatment of HNSCC. In the future, an increasing number of clinical trials are needed.

Although TILs are usually safe, there are potential clinical risks such as on-target off-tumor toxicity, off-target reactivity, and cytokine-release syndrome (Casucci et al., 2015; Rapoport et al., 2015). Therefore, more attention should be given to the safety of its clinical application to avoid complications.

## 8 Epidermal growth factor receptor-targeted therapies for HNSCC

Epidermal growth factor receptor (EGFR) is a prototypical receptor tyrosine kinase that is overexpressed in HNSCC and affects the proliferation, apoptosis, angiogenesis, and metastasis of tumor cells (Nair et al., 2022). Many studies have shown that the overexpression of EGFR in HNSCC directly correlates with worse outcomes (Temam et al., 2007; Agulnik, 2012; Bossi and Platini, 2017). To date, EGFR blockades are attractive targets in HNSCC patients and anti-EGFR strategies such as IgG-based monoclonal antibodies (mAbs), have shown acceptable clinical benefits.

EGFR-targeted mAbs can induce ADCC through Fc receptor-bearing immune cells (Goel et al., 2022). Commonly used mAbs include the following: cetuximab, panitumumab, zalutumumab, and nimotuzumab. Cetuximab is a chimeric monoclonal antibody that binds to domain III of the extracellular region of EGFR and results in apoptosis induction and cancer proliferation and angiogenesis inhibition (Matta and Ralhan, 2009). Cetuximab can inhibit the phosphorylation of EGFR and prevent signals from being transmitted to the cell (Huang and Harari, 2000). In addition, cetuximab can activate NK cells to induce ADCC (Baysal et al., 2021). Panitumumab is a fully human anti-EGFR mAb (Vermorken et al., 2013). Several ongoing phase II studies are currently evaluating panitumumab in locally advanced HNSCC (NCT00547157, NCT00500760, and NCT00798655) or metastatic/recurrent HNSCC (NCT00454779). An ongoing phase III trial is evaluating treatments (panitumumab + radiotherapy vs. cisplatin + radiotherapy) for locally advanced HNSCC (NCT00820248). Zalutumumab is a fully human high-affinity anti-EGFR mAb for advanced, metastatic, and/or unresectable HNSCC (Bleeker et al., 2004). In a phase I/II study in 28 patients with metastatic/recurrent HNSCC, patients treated with zalutumumab were associated with an ORR of 7.1% (Bastholt et al., 2007). Nimotuzumab, as a humanized anti-EGFR mAb, has been granted approval in HNSCC. In a phase I/II trial, nimotuzumab plus radiotherapy was evaluated in 24 patients with locally advanced HNSCC (Crombet et al., 2004). The ORR was 81%, and the three-year OS rate was 66.7% with 200–400 mg nimotuzumab. A double-blind trial involving 17 patients with locally advanced HNSCC was conducted to evaluate the combination of nimotuzumab and concurrent chemotherapy (Rodríguez et al., 2010). Complete ORRs were 59.5% for patients receiving nimotuzumab and radiotherapy versus 34.2% of patients receiving radiotherapy alone.

Recently, there has been significant interest in assessing treatment efficacy with dual inhibition. EGFR inhibition has a large impact on the TME through activation of ADCC *via* NK cells, promoting cross-talk between NK cells and DCs, and priming CTLs (Ferris et al., 2018b). However, these immune related mechanisms lead to negative feedback loops which may limit the efficacy of anti-EGFR mAbs. For example, cetuximab-induced ADCC can stimulate IFN-γ secretion from NK cells, improving NK and DC crosstalk, but it also induces PD-L1 expression and therefore inhibits active T and NK cells, which assists tumor immune escape of HNSCC (Bauman and Ferris, 2014). Thus, simultaneous application of cetuximab and immune checkpoint inhibitors may have synergistic effects to improve patient outcomes. It is worth noting that NK-cell immunity is important for HNSCC immunotherapy and depends on the balance of the interaction of activating and inhibitory receptors on their surface (Carotta, 2016; Bunting et al., 2022). Tumor cells usually decrease the expression of MHC-I to evade T-cell recognition of tumor antigens, and the applicability of T-cell-based immunotherapies needs to gain neoantigens for the induction of adequate responses. By comparison, NK cells can recognize tumor cells independent of their MHC status and require no presentation of neoantigens. Moreover, NK-cell responses can further shape the TME toward activation of the adaptive immunity. Therefore, NK-cell-based immunotherapy combined with anti-EGFR mAbs reestablishes functional NK-cell responses, which can generate more durable antitumor responses.

## 9 Conclusion and outlook

In this paradigm shift in the treatment of HNSCC, the therapeutic potential of immunotherapy is beginning to be recognized in clinical care, but more attention and research are still needed:

(1) Although HNSCC is generally characterized by a high tumor mutational burden, HNSCC is an “immune desert” tumor that can hijack multiple parts of the tumor immunity cycle to evade immune recognition and suppress immune system activation. How to overcome immune escape to maximize HNSCC treatment is an important issue.
(2) The effectiveness of single immunotherapy is limited, and the combination of multiple immunotherapy strategies should be considered. Novel combinations of immunotherapy strategies are critical for improving patient response and combating immune resistance that may occur during treatment. The simultaneous use of multiple immune-targeted agents as a therapeutic strategy has shown promise. Nowadays, identifying appropriate regimens with minimal toxicity and durable responses is the goal of immunotherapy clinical trials. In addition, the combination with conventional radiotherapy and chemotherapy has good prospects. For example, chemotherapy can trigger ICD, which has a synergistic effect with immunotherapy.
(3) The biosafety of immunotherapy requires attention. The toxicities associated with immunotherapy differ from those associated with traditional systemic therapy. Many of these side effects are autoimmune compared to renal failure and anemia, which are seen with standard cytotoxic therapy. Once autoimmune rejection occurs, more serious consequences can occur. Therefore, more preclinical studies are needed to ensure the safety of immunotherapy.
(4) Since few studies have attempted to investigate whether immunotherapy is safe and effective in treating rare subclasses of HNSCC, more evidence is needed to confirm whether patients with these rare subclasses should be treated with immunotherapy.
(5) Vigilant patient evaluation, monitoring, and management strategies are crucial when administering immunotherapies. There are also many management considerations, including biomarker testing prior to immunotherapy administration, when to halt or delay treatment in the event of an immune-related adverse event, and for how long to continue treatment. In addition, the potential quality of life issues pertaining to treating HNSCC with immunotherapies need to be considered.
